# From Light to Energy: Machine Learning Algorithms for Position and Energy Deposition Estimation in Scintillator–SiPM Detectors

**DOI:** 10.3390/s26010101

**Published:** 2025-12-23

**Authors:** Yoav Simhony, Alex Segal, Ofer Amrani, Erez Etzion

**Affiliations:** 1 Raymond and Beverly Sackler School of Physics and Astronomy, Tel Aviv University, Tel Aviv-Yafo 6997801, Israel; yoav.simhony@gmail.com (Y.S.); ereze@tau.ac.il (E.E.); 2 School of Electrical Engineering, Tel Aviv University, Tel Aviv-Yafo 6997801, Israel; ofera@tauex.tau.ac.il; 3 Unit of Mathematics, Afeka College of Engineering, Tel Aviv-Yafo 6998812, Israel

**Keywords:** SSPD, machine learning, gradient boosting

## Abstract

Scintillator-SiPM Particle Detectors (SSPDs) are compact, low-power devices with applications including particle physics, underground tomography, cosmic-ray studies, and space instrumentation. They are based on a prism-shaped scintillator with corner-mounted SiPMs. Previous work has demonstrated that analytic algorithms based on a physical model of light propagation can reconstruct particle impinging positions and tracks and estimate deposited energy and Linear Energy Transfer (LET) with moderate accuracy. In this study, we enhance this approach by applying machine learning (ML) methods, specifically gradient boosting techniques, to improve the accuracy of spatial location and energy deposition estimation. Using the GEANT4 simulation toolkit, we simulated cosmic muons and energetic oxygen ions traversing an SSPD, and we trained XGBoost and LightGBM models to predict particle impinging positions and deposited energy. Both algorithms outperformed the analytic baseline. We further investigated hybrid strategies, including hybrid boosting and probing. While hybrid boosting provided no significant improvement, probing yielded measurable gains in both position and LET estimation. These results suggest that ML-driven reconstruction provides a powerful enhancement to SSPD performance.

## 1. Introduction

The ability to reconstruct charged particle trajectories and energy deposition with compact detectors has significant implications for cosmic-ray studies, spaceborne instrumentation, and applied particle physics. Scintillator detectors instrumented with Silicon Photomultipliers (SiPMs) are particularly attractive due to their compactness, low power consumption, and robustness against environmental conditions. A recent contribution by ref. [1] described the Scintillator–SiPM Particle Detector (SSPD). This detector was introduced in ref. [2] and later validated through in-orbit tests aboard the International Space Station [3]. The SSPD consists of a truncated prism scintillator read out by four SiPMs at its corners. The analytic model for this configuration translated sensor intensity signals into estimates of particle impinging position and energy deposition. By applying an SSPD array, one can achieve particle track reconstruction and Linear Energy Transfer (LET) estimation, resulting in millimeter-scale localization and LET uncertainties of 5–10% under favorable conditions.

Due to the geometrical distribution of the SiPMs and due to some numerical instability of the algorithm described in ref. [1], it underperforms in certain areas of the scintillator, mostly for particle events localized at the extremity of the SSPD.

In parallel, the broader field has increasingly recognized the promise of ML methods for scintillator-based event reconstruction. For example, a 2021 study [4] demonstrated that boosted decision trees and neural networks can outperform analytic techniques in predicting event parameters from scintillation light distributions, particularly in complex or high-noise scenarios. This work demonstrated ML implementations to detector response modeling showing that data-driven methods are capable of capturing effects beyond the reach of analytic formulations. In their work, the authors compared boosting algorithms such as XGBoost, probabilistic neural networks and an analytic model in various geometries and sizes of scintillators in the case of muon particles on a track perpendicular to the scintillator’s surface. They showed that the best results are achieved using a square scintillator and XGboost.

Related research on the application of ML to particle tracking and detector optimization has demonstrated promising results across various detector types. For example, Yaary et al. [5] compared tree-based algorithms and neural networks for enhancing real-time tau-lepton selection in proton–proton collisions. Additional studies have explored similar approaches in diverse contexts, including event reconstruction, position estimation, and particle classification [6,7,8,9,10,11].

Beyond these applications, several works have demonstrated that gradient-boosted decision trees (GBDTs) are highly effective for detector calibration and scintillator-based response modeling. In particular, Müller et al. [12,13] showed that gradient tree boosting provides accurate transverse and depth-of-interaction reconstruction in monolithic scintillator PET detectors. In gamma spectroscopy, XGBoost and LightGBM have been employed for high-precision efficiency calibration of HPGe detectors [14,15]. These studies collectively demonstrate that GBDT models are powerful tools for learning complex detector-response functions, particularly in regimes where analytic models exhibit systematic limitations.

Motivated by these developments, the present study seeks to combine the analytic physics-based algorithm described in ref. [1] with ML algorithms. Specifically, we investigate whether gradient boosting algorithms—XGBoost and LightGBM—can improve track reconstruction and LET estimation in the SSPD configuration of ref. [1]. Beyond direct application of boosting, we also explore hybrid strategies that integrate analytic-physics-based and ML-driven approaches, including hybrid boosting and probing [16]. By comparing analytic, pure ML, and hybrid models, we aim to assess the practical gains in estimation accuracy achievable through ML for SSPDs. We evaluate these methods, similarly to ref. [1], on GEANT4 [17] simulated muon particles and high-energy oxygen ions.

## 2. Methods

### 2.1. Scintillator–SiPM Particle Detector (SSPD)

The detector employed in this study is the Scintillator–SiPM Particle Detector (SSPD), a compact device for charged-particle tracking and energy-loss measurements. The design is based on the configuration described in ref. [1], and is summarized here so that the present manuscript is fully self-contained.

#### 2.1.1. Geometry and Materials

**Figure 1:**
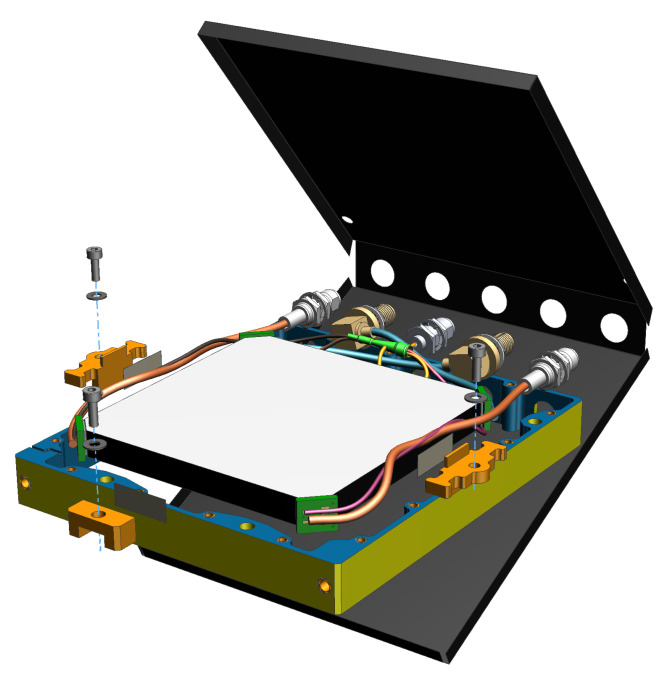
CAD design of an SSPD. Conspicuously depicted is a yellow aluminum frame equipped with five SubMiniature version A (SMA) connectors. Four SensL (Cork, Ireland) FJ-60035 SiPM sensors, colored green, are optically coupled to the truncated corners of an EJ-200 prism-shaped scintillator (Sweetwater, TX, USA) and electrically connected to the SMA connectors. Also visible are the black-coated side faces of the scintillator. This SSPD is subsequently sandwiched by a folded sheet of Thorlabs BKF12 (Newton, NJ, USA) matte black 70 µm aluminum foil.

**Figure 2:**
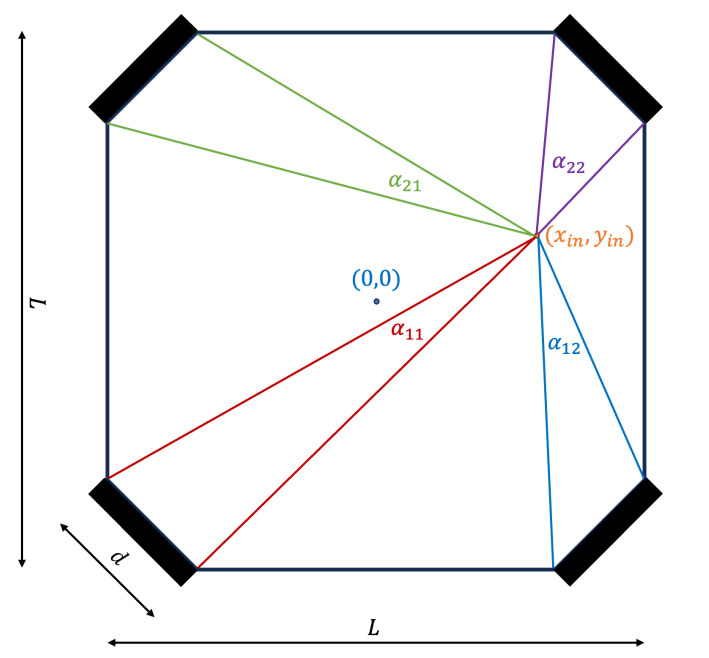
Top view of the SSPD [1]. The four SiPMs are positioned at the truncated corners of a prism-shaped scintillator. A particle impinging on the scintillator at position $\left(x_{in},y_{in}\right)$ is shown, along with the angles $\left(\alpha_{11},\alpha_{12},\alpha_{21},\alpha_{22}\right)$ defined between the impinging point and the edges of the four SiPMs.

The SSPD consists of a polyvinyl toluene (PVT) scintillator machined into a square prism with an active transverse area of $70\times 70\;mm^{2}$ and a thickness of 6.7 mm. To enable efficient readout using only four sensors, each of the four corners of the scintillator is truncated forming flat $6.7\times 6.7\;mm^{2}$ facets. A silicon photomultiplier (SiPM) is optically coupled to each truncated corner, such that the sensors face diagonally toward the scintillator’s interior (see Figure 1 and Figure 2).

The long side faces of the scintillator are coated with a black light-absorbing paint to suppress stray reflections, whereas the top and bottom surfaces are polished to support total internal reflection (TIR). The entire assembly is enclosed in a light-tight housing to prevent ambient light contamination and reduce secondary reflections.

#### 2.1.2. Operating Principle

A charged particle traversing the PVT scintillator excites the material and produces isotropic scintillation photons along its trajectory. The SSPD’s optical system is designed to guide this emitted light within the scintillator through total internal reflection from the top and bottom surfaces. Light that reaches the SiPMs is detected, while stray light is almost entirely absorbed, thereby not influencing the SiPMs’ measurements.

Consequently, the relative photon counts recorded by the four sensors encode the two-dimensional impact point of the particle. Positions closer to a given corner yield a larger signal in the corresponding SiPM, enabling a triangulation-like reconstruction of the (x,y) coordinates. The total number of absorbed photons is proportional to the deposited energy, and is thus correlated with the particle’s Linear Energy Transfer (LET).

#### 2.1.3. Simulation

We generated two datasets using GEANT4 (version 10.7):

- Muon dataset: $10^{5}$ simulated 4 GeV muons with randomized incidence positions and angles.
- Oxygen dataset: $10^{5}$ simulated 18 GeV oxygen ions with randomized incidence positions and angles.

For each simulated event, we recorded the number of photons measured by each SiPM (photon vector), the particle’s energy deposition along the track within the scintillator, and the location of incidence.

The impact points are generated uniformly over the scintillator surface in GEANT4. In a post-processing step, we select only events whose entry point lies within a central square of size 70 × 70 mm^2^, so the resulting (x,y) labels form a dense, approximately uniform point cloud within that region. We then randomly partition the events into training and test subsets using an 80/20 split at the event level; individual coordinates do not repeat exactly between train and test sets, because they are continuous quantities drawn from a uniform distribution.

In the GEANT4 simulation, each event contains the coordinates of the particle’s entry and exit points on the scintillator surface. We define the true impact point as the midpoint between these two coordinates:

(1) $$\left(x_{\mathrm{true}},\;y_{\mathrm{true}}\right)\;=\;\frac{1}{2}\;[\left(x_{\mathrm{enter}},y_{\mathrm{enter}}\right)+\left(x_{\mathrm{exit}},y_{\mathrm{exit}}\right)].$$

This definition is appropriate for thin scintillators, where transverse displacement within the material is negligible. The analytic reconstruction, ML and hybrid algorithms use as input the four quantities measured by the SiPMs, directly provided by GEANT4. As detailed in ref. [1], real-world calibration measurements using cosmic muons were used to determine the SiPM response coefficients; these calibrated constants would also be used when applying the ML and hybrid reconstruction to real data.

### 2.2. The Physics-Based Analytic Model

The physics-based analytic model (AM) introduced in ref. [1] is based on the fact that the number of photons reaching each SiPM is proportional to the angle $\alpha_{ij}$ between the edges of the SiPM and the particle incidence location ($\left(\alpha_{11},\alpha_{12},\alpha_{21},\alpha_{22}\right)$ in Figure 2):

(2) $$N=C_{s}\;\cdot \;\mathrm{LET}\;\cdot \;\frac{\alpha }{2\pi },$$

where LET is the particle’s linear energy transfer and $C_{s}$ is a constant dependent on the scintillator’s geometry, density and efficiency in converting particle deposited energy to electromagnetic energy (photons).

Using this model, one may pre-compute the resulting measurements (up to a multiplicative constant) on a grid with a given resolution, and use the obtained results to estimate the particle’s impinging location. For details see Algorithm 1 in ref. [1]. After the particle’s impinging location is obtained, the particle’s LET may be approximated using the analytic model.

The analytic approach is computationally efficient and physically interpretable, making it suitable for deployment in resource-constrained environments such as space missions. However, due to numerical instability of the algorithm, nonlinearities in events where the particle impinges the scintillator very close to a SiPM, and due to the variability of the Geometric Dilution of Precision (GDOP) across the SSPD’s area, it under-performs at certain areas of the scintillator, mostly near the edges, where the GDOP is small (see discussion in the results section).

## 3. Machine Learning Methods

### 3.1. Gradient Boosted Regression (XGBoost and LightGBM)

We first explore direct regression from photon vectors to incidence location using gradient boosting. Two widely used frameworks are considered:

- XGBoost is a highly effective tree boosting algorithm [18]. It works by constructing additive ensembles of decision trees using second-order gradient information. It is robust to nonlinear feature interactions and performs well on structured datasets. Since its introduction, XGBoost, due to its scalability and speed, quickly became one of the most popular ML algorithms. In our context, it effectively models the nonlinear relationship between the signals detected by the SiPMs, the particle impinging positions and as a result, LET estimation.
- Designed by Microsoft, LightGBM [19] introduces histogram-based training and leaf-wise tree growth to improve training and prediction efficiency. LightGBM is designed for faster training speeds and higher efficiency, particularly on large datasets, compared to some other gradient boosting frameworks like XGBoost. This is achieved through innovative techniques such as Gradient-based One-Side Sampling (GOSS) and Exclusive Feature Bundling (EFB), which optimize the process of finding optimal split points in decision trees.

XGBoost and LightGBM were selected because they represent state-of-the-art gradient boosting methods, well suited to capturing the nonlinear mapping between scintillation light distribution and particle impact parameters. XGBoost is robust and widely validated, while LightGBM offers improved training efficiency on large datasets. Using both models provides a robust methodological basis for evaluating gradient boosting performance in this context. It is worth noting that both methods improve the localization accuracy compared to the analytical baseline. However, they replace rather than integrate with the physics model, potentially losing interpretability and extrapolation power in regimes outside the training distribution.

### 3.2. Hybrid Intelligence: A Fusion of Machine Learning and Analytics

To combine the advantages of the analytic model with those of ML algorithms, we examined two established approaches for hybrid architectures (see ref. [16]).

#### 3.2.1. Boosting

**Figure 3:**
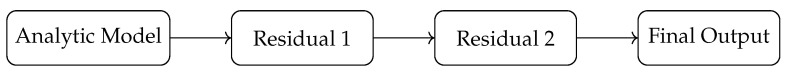
Compact schematic of the residual-boosting hybrid model.

Boosting is a strategy that uses the analytic model as a base predictor, and corrects it iteratively by learning the residual errors. Specifically, a sequence of regressors $\Gamma_{m}$ is trained to predict residuals between the analytic estimate and the ground truth. These regressors, which may be of different types, are stacked in a chain, and each regressor attempts to minimize the error of the previous element in the chain (see Figure 3). Their weighted outputs are added back to the analytic prediction:

(3) $$\hat{y}=AM(X)+\sum_{m=1}^{M}\beta_{m}\;\cdot \;\Gamma_{m}\left(X\right),$$

where $\beta_{m}$ are optimized coefficients that are trained to minimize the error up to the *m*-step. For the full algorithm, see Algorithm 2 in ref. [16]. This residual boosting approach systematically reduces bias across the detector while preserving the physical basis of the analytic model. Notice that the model is independent of the type of regressors $\Gamma_{m}$, which can be chosen at will. We have tested Boosting with both—XGBoost and LightGBM.

#### 3.2.2. Probing

**Figure 4:**
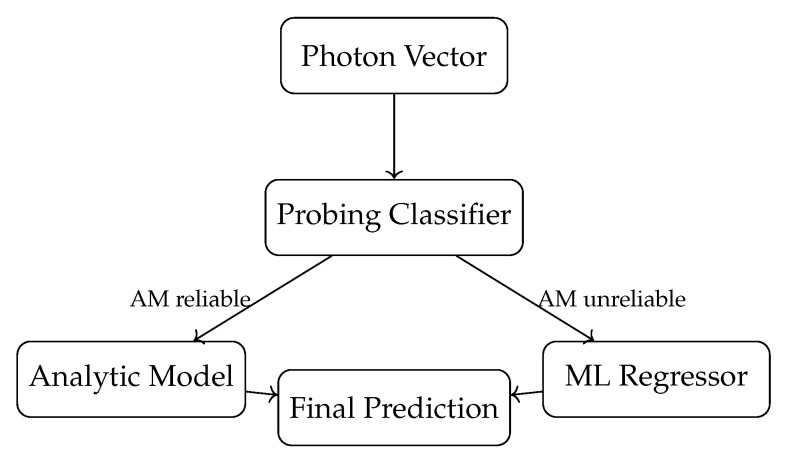
Compact schematic of the Probing hybrid reconstruction architecture.

Probing is a hybrid strategy well-suited for scenarios where an analytic model offers fast but occasionally inaccurate predictions. A binary classifier is trained to predict whether the analytic model’s error for a given input is below a threshold τ. At inference, if the binary classifier predicts high accuracy, the analytic model is used for prediction; otherwise, the event is routed to an ML regressor (e.g., LightGBM/XGboost). This selective strategy (see Figure 4) retains the speed and physics grounding of the analytic method across much of the detector while deploying ML only in challenging regions such as edges and corners. For full details, see Algorithm 3 in ref. [16].

In our work, we have tested both of these approaches, with both types or regressors. Some showed a significant improvement, and some did not.

To clarify the rationale behind the hybrid methods, we note that both Boosting and Probing are designed to address complementary limitations of the analytic model. Residual boosting operates under the theoretical assumption that the analytic model captures the dominant first-order light-sharing behavior, while the machine-learning regressors learn higher-order corrections that arise from nonlinear optical effects, geometric distortions, and SiPM response variations. Probing, in contrast, follows a model-selection logic; an auxiliary classifier predicts whether the analytic model is reliable for a given photon pattern, thereby routing each event either to the analytic solution or to a machine-learning regressor. This selective mechanism exploits the observation that the analytic model is highly accurate in the central region of the scintillator but exhibits systematic biases near edges and corners.

## 4. Training and Hyperparameters

### 4.1. Inputs and Targets

The feature vector per event is the 4-tuple measurement of photons reaching the four SiPMs (Top-Left, Bottom-Right, Bottom-Left, Top-Right). The model’s target is the 2D particle impinging position within the scintillator, defined at the midpoint along the Z-axis. Prior to any ML stage (regression or classification), inputs are L2-normalized across features. The data was split 80–20% for training and testing, with cross-validation.

### 4.2. XGBoost and LightGBM

The XGB regressor was trained for optimizing the root mean squared error (RMSE) with $10^{5}$ estimators, a small learning rate of $2^{-4}$ and a maximal depth of 4. Early stopping was not used.

The LightGBM regressor was trained with a max depth of 7, learning rate of  0.01 and roughly 1000 estimators.

To ensure that the gradient-boosted decision tree (GBDT) models used in this work (LightGBM and XGBoost) operate near optimal settings, we carried out a dedicated hyperparameter optimization study using the Optuna framework [20]. Optuna implements a Bayesian optimization strategy based on Tree-structured Parzen Estimators (TPEs), which enables efficient exploration of high-dimensional search spaces with minimal manual intervention.

#### 4.2.1. Search Space

For both LightGBM and XGBoost regressors, we defined a search space that spans a broad range of model complexities, learning rates, and sampling strategies. The following hyperparameters were included in the study:

- maximum tree depth: d∈[3,15];
- number of leaves (LightGBM): $n_{\mathrm{leaves}}\in \left[16,256\right]$;
- learning rate: η∈[0.001,0.3];
- number of boosting rounds: $N_{\mathrm{est}}\in \left[500,5000\right]$;
- subsampling ratio: subsample∈[0.5,1.0];
- feature sampling ratio: colsample_bytree∈[0.5,1.0];
- minimum child samples (LightGBM): $n_{\mathrm{child}}\in \left[1,100\right]$.

#### 4.2.2. Objective Function

Each trial trains a model on the training subset and evaluates its performance on a validation subset using the mean squared error (MSE):

$$L=\frac{1}{N}\sum_{i=1}^{N}{∥{\hat{y}}_{i}-y_{i}∥}_{2}^{2}.$$

The objective of the optimization is to minimize this validation loss. We performed between 50 and 100 trials per scan, which we found sufficient for convergence.

#### 4.2.3. Best-Performing Configurations

Optuna consistently identified models with moderate depth, intermediate learning rates, and relatively aggressive subsampling as the best candidates. For LightGBM, the top-performing configuration typically included

$$d\approx 7–11,\;n_{\mathrm{leaves}}\approx 80–180,\;\eta \approx 0.01–0.05,$$

with subsample and feature-subsample ratios near 0.7–0.9.

#### 4.2.4. Hyperparameter Importance

The hyperparameter importance analysis performed with Optuna shows that model performance is dominated by the tree depth parameter (max_depth, 76% of the explained variance) and, to a lesser extent, by the feature subsampling ratio (colsample_bytree, 20%). All other parameters contributed less than 2% to the observed variation in validation error within the scanned ranges, indicating that the reconstruction task is relatively insensitive to learning rate, number of estimators, and leaf-related regularization. This suggests that model expressiveness, rather than learning rate or training strategy, is the primary factor controlling accuracy on this dataset.

### 4.3. Convergence Diagnostics

**Figure 5:**
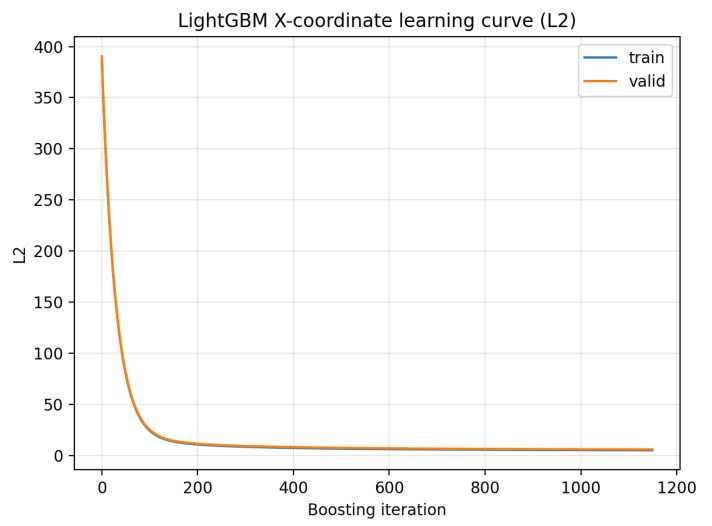
Learning curves for the gradient-boosted decision tree models. Training and validation losses evolve almost identically, demonstrating stable convergence and the absence of overfitting.

To verify that the gradient-boosted decision tree models converged properly and to document their generalization behavior, we include training and validation learning curves for LightGBM (Figure 5). These curves show the loss on the training and held-out validation sets as a function of the boosting iteration. As expected for the GEANT4-simulated dataset, the curves are nearly identical, indicating stable convergence without overfitting.

### 4.4. Hybrid Boosting

Hybrid boosting, also called iterative residue training, was implemented and tested with up to 25 residual learners $\Gamma_{m}$ according to Formula (3), and the multiplicative coefficients were optimized according to the algorithm in ref. [16].

### 4.5. Probing Hybrid

A classifier was trained that predicts whether the error of the analytic model is likely to be below a threshold τ. We tested XGBoost, LightGBM and kNN classifiers. The kNN classifier with k=2 and Manhattan distance performed best. Using this classifier, we implemented the hybrid model that applies the analytic model if the estimated error is below τ and the ML method otherwise. The threshold τ was chosen for the optimal results using grid search.

## 5. Results

### 5.1. Localization and LET Estimation Accuracy

**Figure 6:**
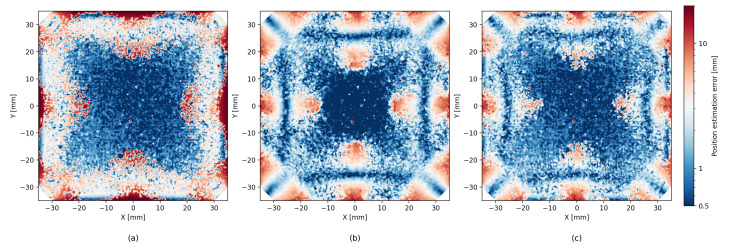
Comparison of localization error heatmaps for the (**a**) analytic model, (**b**) pure machine learning, and (**c**) hybrid approach. Each subfigure uses the same color scale to facilitate direct comparison.

**Figure 7:**
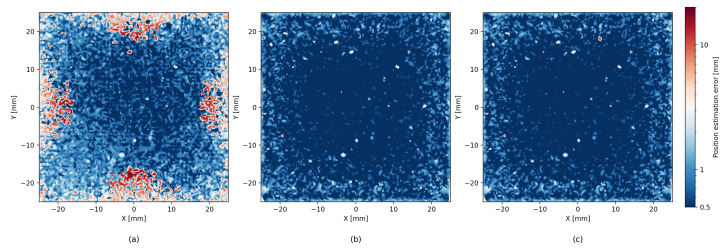
Comparison of localization error heatmaps in a centered 50 mm × 50 mm area of interest of the SSPD for (**a**) the analytic model, (**b**) pure machine learning using LightGBM, and (**c**) hybrid approach with LightGBM. Each subfigure uses the same color scale to facilitate direct comparison.

With the new, larger dataset, the analytic model achieved results comparable to the results in ref. [1], as expected. The ML methods, both XGBoost and LightGBM, provided a significant improvement in accuracy of roughly 30% RMSE over the analytical model. The hybrid method of iterative residue learning did not improve upon the pure ML methods; however, the addition of probing improved the accuracy by another 10% (see Figure 6). The complete results are detailed in Table 1 and Table 2. If we limit the particle interactions with the detector to a centered 50 mm × 50 mm region of interest, the performance improves substantially, with the ML model yielding an average localization error of 0.52 mm, compared to 1.66 mm obtained with the analytic model (see Figure 7).

### 5.2. Error Heatmaps

**Figure 8:**
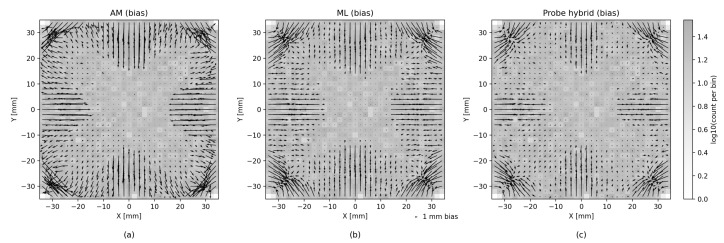
Mean error direction and size vector maps for the (**a**) analytic, (**b**) ML and (**c**) the hybrid models. Notice that the errors are distributed differently on each axis, depending on the location. This difference is due to variability of GDOP across the area of the scintillator and nonlinear effects very close to the SiPMs.

**Figure 9:**
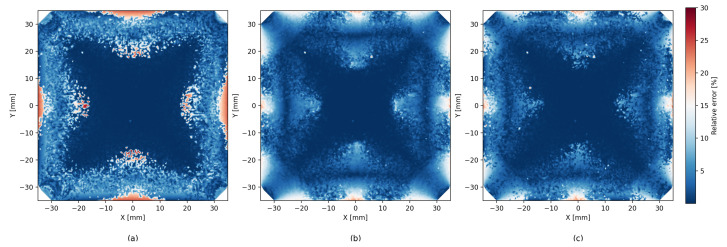
Comparison of relative LET error heatmaps for the analytic model (**a**), pure machine learning (**b**), and hybrid approach (**c**). Each subfigure uses the same color scale to facilitate direct comparison.

Figure 6 shows that the precision of the analytic model is degraded near the edges of the scintillator. Although ML methods help reduce this effect, they tend to produce less accurate estimates near the center area of the scintillator. The hybrid method seems to successfully combine both the analytic and the ML approaches, and we can see an improved estimate near the center as well as near the edges. In addition to the error heatmap, similar effects can be seen in Figure 8, which shows the average error size and the direction map with different models. A similar effect, though to a lesser degree, can be seen for LET estimates in Figure 9.

**Figure 10:**
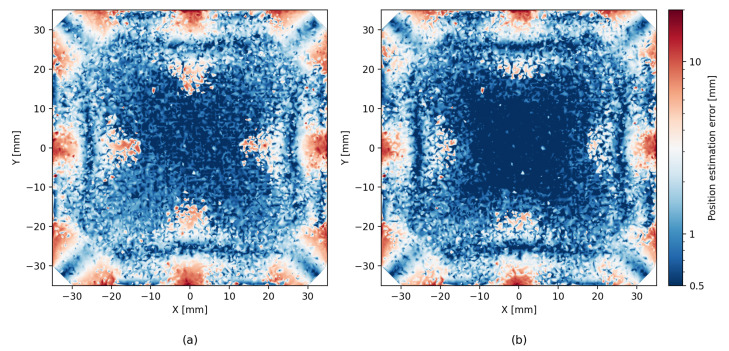
(**b**) Localization error heatmap for the hybrid probing model assuming a theoretical perfect classifier, i.e., a classifier that always selects the model yielding the most accurate estimate for each measurement. For convenience, subfigure (**a**) reproduces the localization error map already shown in Figure 6.

In the probing hybrid architecture, the total reconstruction uncertainty contains two components: the classifier decision uncertainty and the regression uncertainty of the model selected (analytic or ML). While this raises a natural concern that misclassification may amplify prediction error, our results show that this contribution is small. As demonstrated in Figure 10, the performance of an idealized “perfect classifier” (oracle) that always selects the better of the two models is only marginally better (RMSE of 1.6 mm) than that of the implemented probing hybrid. This close agreement indicates that the classifier rarely makes harmful decisions and that classifier-induced uncertainty is negligible compared to the intrinsic prediction uncertainties of the underlying models.

**Figure 11:**
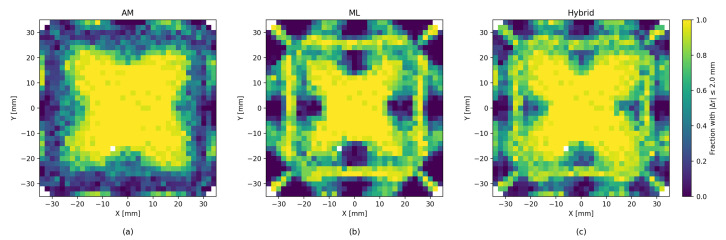
Confusion-matrix-like visualization of local reconstruction performance. The scintillator surface is discretized into $2\times 2\;{\mathrm{mm}}^{2}$ bins according to the *true* interaction position. For each bin we show the fraction of “correct” events, defined as those with a reconstruction error |Δr|≤2mm. This binned accuracy map highlights spatial regions where each method (Analytic Model (**a**), ML regressors (**b**), Hybrid (**c**)) performs well or exhibits systematic degradation.

Beyond global metrics such as RMSE, we also evaluate the spatial structure of reconstruction quality by partitioning the scintillator surface into $2\times 2\;{\mathrm{mm}}^{2}$ bins. Within each bin we compute the fraction of *correct* events, defined as those whose reconstructed position lies within 2mm of the true interaction point. This produces a confusion-matrix-like spatial map of local accuracy, revealing regions where the analytic, ML, or hybrid methods perform consistently well or systematically struggle (see Figure 11). As expected, the patterns are similar to the patters of the error heatmaps presented in Figure 6.

### 5.3. Error Distribution

Error heatmaps reveal that analytic residuals grow near the scintillator’s edges and corners, consistent with the GDOP distribution. ML reduces errors globally but is prone to variance. The probing method suppresses edge artifacts by routing to ML, while the boosting hybrid smooths systematic deviations across the entire surface.

### 5.4. Statistical Analysis

To quantify the robustness of the reconstruction performance and assess the statistical significance of the improvements obtained with the ML and hybrid approaches, we performed a detailed bootstrap analysis of the per-event localization errors on the held-out test set (see Table 3 for summary). The analytic model exhibits a mean error of 3.3 mm with a 95% confidence interval (CI) of [3.25,3.35]mm. The ML regressor achieves a substantially lower mean error of 2.275mm, with CI [2.246,2.303]mm. The hybrid model provides the best performance, yielding a mean error of 1.913mm with CI [1.887,1.94]mm.

To compare methods directly, we computed paired confidence intervals for the differences in mean error. The improvement of the ML model over the analytic baseline is $\Delta {\bar{e}}_{\mathrm{Analytic}-\mathrm{ML}}=1.025\;\mathrm{mm}$, with a 95% CI of [0.98,1.072]mm. The improvement of the hybrid model over the analytic model is even larger, $\Delta {\bar{e}}_{\mathrm{Analytic}-\mathrm{Hybrid}}=1.387\;\mathrm{mm}$, CI [1.345,1.429]mm. The difference between the ML-only and hybrid methods is also statistically significant, with $\Delta {\bar{e}}_{\mathrm{ML}-\mathrm{Hybrid}}=0.362\;\mathrm{mm}$, CI [0.329,0.378]mm. In all three comparisons, the 95% confidence intervals exclude zero, demonstrating that the performance gains of the ML and hybrid approaches over the analytic baseline, and the gain of the hybrid method over the ML approach are statistically significant at the 95% level.

We additionally performed a statistical analysis restricted to the central $50\times 50\;{\mathrm{mm}}^{2}$ effective area of the scintillator, where light-sharing patterns are more symmetric and edge effects are suppressed. In this region, the analytic model achieves a mean localization error of 1.627 mm (SEM), with a 95% confidence interval (CI) of [1.580,1.673]mm. The ML-only model shows a substantial improvement, with a mean error of 0.501mm, CI [0.493,0.509]mm. The hybrid model reaches a nearly identical performance level, yielding a mean error of 0.499±0.004mm and CI [0.492,0.507]mm.

The improvement of the ML model over the analytic baseline is $\Delta {\bar{e}}_{\mathrm{Analytic}-\mathrm{ML}}=1.126\;\mathrm{mm}$, with a 95% CI of [1.080,1.171]mm. In contrast, the difference between the ML and hybrid methods was statistically insignificant.

## 6. Discussion and Conclusions

Recent studies have shown that very high spatial resolution can be achieved in plastic scintillators when using dense photodetector coverage. For example, a 3 cm × 3 cm tile instrumented with sixteen SiPMs on one face achieved sub-millimeter resolution under beam-test conditions [21]. Such performance, however, relies on a drastically different design philosophy: small scintillator tiles with high SiPM fill factor, high power consumption, and increased readout complexity. In contrast, the SSPD concept targets a sparse-readout, large-area, low-power detector suitable for spaceborne and cosmic-ray applications, where only four corner-mounted SiPMs are available, while providing comparable performance.

The physical and geometric model presented is this paper is a first-order approximation, which provides a strong ground for improvement by applying ML models as a replacement or addition to the analytic one.

We extended the SSPD analytic framework by integrating ML and hybrid approaches, achieving a localization accuracy of ∼2.0 mm (RMSE) and robust LET estimation with 98% accuracy for high-energy oxygen ions impinging on a detector with dimensions of 70 mm × 70 mm ×6.7 mm, across a range of incidence positions and angles. For muons we have achieved ∼4.0 mm RMSE with LET estimation accuracy of 96%. These results suggest that hybrid physics+ML methods offer a strong candidate for future particle detectors.

Each reconstruction strategy offers distinct advantages and limitations. The analytic model is interpretable, fast, and grounded in photon transport physics, making it ideal for deployment on resource-limited hardware. Its weakness lies in reduced accuracy in regions where the light-sharing geometry provides limited discriminating power. XGBoost offers strong modeling of nonlinearities, but is computationally heavier and less efficient for large-scale data. LightGBM provides faster training and better scalability, with slightly improved results compared to XGBoost. In our tests, this performance gain was verified empirically, confirming that LightGBM trains significantly faster for the considered datasets.

Beyond the numerical improvements demonstrated in this work, it is instructive to examine the qualitative behavior of the analytic, ML, and hybrid models. The analytic model produces reliable predictions in the central region of the scintillator, while ML regressors offer improved adaptability in regions where small variations in photon patterns might entail meaningful positional shifts. The hybrid model combines these strengths; the boosting variant learns systematic corrections to the analytic estimate, whereas the probing strategy selectively applies the analytic or ML prediction depending on expected local reliability.

From an application perspective, the hybrid strategy is particularly suited for compact, low-power detector systems where increasing readout channels is impractical. Its main limitations stem from the intrinsic information content of four-channel readout; reconstruction accuracy is ultimately bounded by sparse sampling of the scintillation light field. The methods presented in this paper provide a scalable path to improved performance without additional hardware and is expected to remain robust when extended to real data that include optical imperfections, thermal variations, and electrical noise.

Unlike previous studies that achieved sub-millimeter accuracy using dense SiPM arrays or monolithic PET crystals, our work demonstrates competitive performance with only four SiPMs in a sparse-readout SSPD configuration. Prior approaches rely solely on either analytic models or pure ML methods, whereas our hybrid framework systematically integrates a physics-based light-sharing model with machine-learning corrections and dynamic selection. This design not only improves accuracy and robustness near scintillator edges, where analytic models suffer from GDOP limitations, but further offers a scalable, low-power solution most suitable for spaceborne and resource-constrained applications.

## Figures and Tables

**Table 1:** Performance summary on Geant4 muon simulations.

Method	Position RMSE [mm]		Mean LET Error	
	Full Area	50 × 50	Full Area	50 × 50
Analytic (AM)	6.1	5	6%	4%
LightGBM	4.3	2	4.5%	1%
XGBoost	4.2	2.05	4.5%	1%
Probing LightGBM	4.0	2	4%	1%
Probing XGBoost	4.0	2	4%	1%

**Table 2:** Performance summary on Geant4 oxygen simulations.

Method	Position RMSE [mm]		Mean LET Error	
	Full Area	50 × 50	Full Area	50 × 50
Analytic (AM)	3.3	1.6	4%	2%
LightGBM	2.3	0.52	3%	0.3%
XGBoost	2.3	0.53	3%	0.25%
Probing LightGBM	1.9	0.51	2.5%	0.3%
Probing XGBoost	2	0.51	2.5%	0.25%

**Table 3:** Statistical analysis of localization error for the analytic, ML, and hybrid models. Reported values include mean error and 95% confidence intervals (CIs).

Full Detector Area		
**Model**	**Mean Error [mm]**	**95% CI [mm]**
Analytic Model	3.300	[3.25, 3.35]
ML only	2.275	[2.246, 2.303]
Hybrid	1.913	[1.887, 1.940]
**Comparison**	**$\Delta \bar{e}$ [mm]**	**95% CI [mm]**
Analytic − ML	1.025	[0.98, 1.072]
Analytic − Hybrid	1.387	[1.345, 1.429]
ML − Hybrid	0.362	[0.329, 0.378]
**Central $50\times 50\;{\mathrm{mm}}^{2}$ Region**		
**Model**	**Mean Error [mm]**	**95% CI [mm]**
Analytic Model	1.627	[1.580, 1.673]
ML only	0.501	[0.493, 0.509]
Hybrid	0.499	[0.492, 0.507]
**Comparison**	**$\Delta \bar{e}$ [mm]**	**95% CI [mm]**
Analytic − ML	1.126	[1.080, 1.171]
ML − Hybrid	0.002	*not significant*

## Data Availability

The data presented in this study are available on request from the corresponding author.
